# Metabolic Reprogramming of Microglia in Neuroinflammation and Depression

**DOI:** 10.3390/ijms27093984

**Published:** 2026-04-29

**Authors:** Qingru Wu, Jing Tian, Yan Gu, Xiaoying Bi, Hailing Zhang

**Affiliations:** 1 Department of Neurology, Changhai Hospital, Naval Medical University, Shanghai 200433, China; wuqingru0111@smmu.edu.cn (Q.W.); tianjing331@126.com (J.T.); 2 National Key Laboratory of Immunity and Inflammation, Institute of Immunology, Naval Medical University, Shanghai 200433, China; guyan@immunol.org

**Keywords:** depression, microglia, immunometabolism, metabolic reprogramming, neuroinflammation, glycolysis, lipid metabolism, glutamate, mitochondria

## Abstract

Depression is a highly heterogeneous psychiatric disorder with its pathogenesis increasingly linked to dysregulated neuroinflammation. Microglia, as the resident immune cells of the central nervous system (CNS), play a pivotal role in the initiation and progression of the neuroinflammation and the pathophysiology of depression. These cells exhibit a dual role in pro- and anti-inflammatory processes, dynamically regulating immune responses through immunometabolic reprogramming in response to environmental cues. This review elaborates how metabolic remodeling in microglia, particularly within glucose, lipid, and amino acid pathways, drives their polarization toward a pro-inflammatory phenotype. This shift promotes depression pathogenesis via the release of inflammatory factors, disruption of synaptic plasticity, and mediation of neurotoxicity. We further discuss the impact of existing antidepressants on cellular metabolism and highlight the promise and challenges of targeting specific microglial metabolic pathways as a novel therapeutic strategy. This synthesis provides new insights into the immunometabolic mechanisms of depression and outlines directions for developing targeted treatments.

## 1. Introduction

Depression represents one of the most prevalent and disabling mental and physical health conditions worldwide. Its core clinical features encompass persistent low mood, anhedonia and suicidal behavior [1]. In China, the lifetime prevalence of depressive disorders is 6.8%, with a majority of affected individuals experiencing severe psychosocial impairment [2]. Current pathophysiological models highlight disruptions in monoamine neurotransmission, dysregulation of the hypothalamic-pituitary-adrenal (HPA) axis, impaired neurogenesis, and hyperactivation of inflammatory pathways [3]. A defining characteristic of depression is its pronounced heterogeneity, reflected in highly variable treatment responses: whereas some patients achieve remission with first-line therapies, a significant subset displays substantial antidepressant resistance and suffers lifelong recurrence [4]. Therefore, unraveling the underlying mechanisms and developmental pathways of depression remains an urgent priority.

Microglia, the resident immune cells of the central nervous system (CNS), are essential for immune surveillance and CNS homeostasis [5]. They are actively involved in phagocytosis, synaptic pruning, neuronal protection, and cytokine release [6]. Substantial evidence now links microglial dysregulation to the pathophysiology of depression. Activated microglia produce pro-inflammatory cytokines, chemokines, and reactive oxygen species (ROS), disrupting the neural microenvironment and altering the activity of neurons and glial cells [7,8].

Immunometabolic reprogramming marked by shifts in energy metabolism, biomass synthesis, and the epigenetic landscape critically regulates inflammatory activation across innate and adaptive immune populations [9]. In microglia, a hallmark of the pro-inflammatory state is a metabolic switch from oxidative phosphorylation (OXPHOS) to aerobic glycolysis for adenosine triphosphate (ATP) generation, accompanied by adaptations in the pentose phosphate pathway, fatty acid oxidation, and glutamate metabolism [10]. Microglia express nearly the entire repertoire of metabolic genes, and their functional metabolic profile is shaped by substrate availability and pathway engagement in a state-dependent manner [11]. Previous studies have explored the mechanism and therapeutic value of microglial metabolic reprogramming in diverse neurological pathological contexts [10,12,13,14], but its role in depression remains less defined. Elucidating how shifts in microglial metabolic pathways and metabolite output drive phenotypic changes and sustain neuroinflammation could uncover novel insights and targets for depression.

This review synthesizes current understanding of microglial contributions to depression pathophysiology. We elucidate the mechanisms by which microglia drive neuroinflammation and modulate depressive-like behaviors, with a focused analysis of how metabolic reprogramming and associated metabolites shape their phenotypic and functional states. Furthermore, we evaluate the effects of antidepressant agents on cellular metabolism and highlight emerging therapeutic strategies that target microglial metabolic pathways for the treatment of depression.

## 2. Microglial Function in Depression

In contrast to neurons and other glial cells derived from the neuroectoderm, microglia represent the resident macrophages of the central nervous system and belong to the mononuclear phagocyte system [15]. Under homeostatic conditions, microglia have small soma and ramified protrusions, remaining highly dynamic and monitoring the local microenvironment [16,17]. Upon detection of abnormal proteins, cellular debris, pathogens, or tissue damage within the CNS, surveilling microglia rapidly respond by undergoing morphological changes, proliferating, and acquiring phagocytic capability. Historically, activated microglia were categorized into a simplified binary model of “M1” and “M2” polarized states to describe distinct inflammatory responses [18]. Classically, activated M1 microglia release pro-inflammatory cytokines such as IL-1β, IL-18, and TNF-α, which contribute to neuroinflammation and clearance of harmful substances but may also induce neurotoxicity and tissue damage. In contrast, M2 microglia secrete anti-inflammatory mediators including IL-4, IL-8, and TGF-β, which promote tissue repair and inflammation resolution [19]. However, recent single-cell technologies have revealed that this dichotomous classification is oversimplified, as microglial activation exists along a phenotypic continuum encompassing a spectrum of states with diverse molecular markers and receptor profiles [20]. In models of neurodegenerative diseases such as Alzheimer’s disease (AD), single-cell RNA sequencing (scRNA-seq) has identified a unique disease-associated microglia (DAM) state characterized by the upregulation of genes related to lysosomes, lipid metabolism, and inflammatory responses, which extends beyond the classical M1/M2 framework [21]. Furthermore, in models of chronic stress and depression, scRNA-seq studies have also revealed complex microglial transcriptional changes that cannot be categorized into fixed phenotypes, underscoring the necessity to comprehend the dynamic changes and functional states of microglia [22,23]. In the context of chronic stress and mood disorders, this understanding may contribute to elucidating pathogenic mechanisms and developing interventions.

Depression is increasingly recognized as a microglial disorder [24]. Substantial evidence links depressive onset to aberrant microglial proliferation and functional impairment. Postmortem analyses show elevated expression of microglial markers such as CX3CR1 and TMEM119 mRNA in brain regions including the medial frontal gyrus, superior temporal gyrus, thalamus, and subventricular zone of individuals with depression [25]. Microglial hyperproliferation in the dorsolateral prefrontal cortex (dlPFC), anterior cingulate cortex (ACC), mediodorsal thalamus, and hippocampus has been associated with increased suicide risk in major depressive disorder (MDD) patients [26]. Neuroimaging studies using [^18^F] FEPPA consistently reveal an increased translocator protein (TSPO) distribution volume, indicative of microglial activation, in the prefrontal, cingulate, hippocampal, and insular regions of MDD subjects [27,28,29,30]. Notably, these elevations often normalize following antidepressant treatment, supporting a neuroinflammatory model of depression. Paradoxically, some studies report a non-inflammatory microglial signature in MDD, marked by upregulated homeostatic genes and altered non-inflammatory activity, suggesting complex cellular adaptations over the disease course [22,25,31].

Animal models corroborate the complex involvement of microglial dynamics in depression-related pathology. Chronic stress paradigms, including chronic unpredictable stress (CUS), chronic restraint stress (CRS), and chronic social defeat stress (CSDS), typically reduce microglial density, process complexity, and activation markers. In contrast, early social isolation triggers premature microglial activation and apoptosis in the hippocampus, concomitant with depressive-like behaviors that are reversible upon microglial inhibition by minocycline or enhancement via the macrophage colony-stimulating factor [32,33]. Lipopolysaccharide (LPS)-challenge models further reveal that microglial proliferation can induce neuroinflammation, synaptic deficits, and depression-like behavior, underscoring the context-dependent nature of microglial influence [34,35]. Acute and chronic social defeat stress elicit microglial proliferation in the former and enhanced phagocytosis in the latter [36]. Direct microglial manipulation also modulates depressive phenotypes. *Cx3cr1* knockout exacerbates stress-induced hyper-ramification and impairs antidepressant efficacy [37]. However, it is important to note that animal models, which are typically constructed based on a single, homogeneous etiology, may not fully recapitulate the complex physiological changes observed in the clinical manifestation of diseases. Moreover, behavioral assessments in animals may not accurately correspond to the emotion and cognition symptoms unique to humans. These findings indicate that the microglia–depression relationship is bidirectional and state-dependent, involving context-specific shifts in proliferation, apoptosis, activation, and homeostatic disruption that may align with particular disease stages or subtypes. A deeper functional mapping of microglial states will thus be essential to unravel the pathophysiology of depression.

Microglia contribute to depression pathogenesis largely through their central role in regulating neuroinflammation. A robust body of evidence links psychiatric conditions to chronic inflammatory states [38,39]. Elevated pro-inflammatory cytokines and chemokines such as IL-6, IL-10, TNF-α, and CCL2 are consistently observed in the blood and cerebrospinal fluid of depressed patients, with levels often predictive of antidepressant response [40,41]. Psychosocial stressors can further amplify neuroinflammatory signaling, thereby promoting depressive-like behaviors [42]. Neuroinflammation is a key factor in the pathogenesis of depression, and microglia are the primary mediators of central nervous system inflammation. Firstly, microglia-derived inflammatory mediators directly change neuron function. For example, IL-6 and IL-18 from activated microglia trigger neural stem cell apoptosis and impede neurogenesis, heightening stress susceptibility in mice [43,44], while IL-4-driven Arg1^+^ microglia enhance the hippocampal brain-derived neurotrophic factor (BDNF) to support neurogenesis and stress resilience [45]. IL-6 also acts alongside cyclooxygenase-1 to facilitate prostaglandin synthesis, dampening striatal neuronal excitability and potentially contributing to negative affective states [46]. Additionally, inflammation directly affects mood regulation by impairing neurotransmitter systems. Inflammation upregulates indoleamine 2,3-dioxygenase (IDO), diverting tryptophan metabolism toward the kynurenine pathway. This shift reduces serotonin availability and increases neurotoxic metabolite quinolinic acid [47]. Also, microglia reshape synaptic architecture and neural circuit function through their phagocytic activity. In response to stress or peripheral inflammation, microglia can aberrantly prune synaptic spines and clear myelin, processes essential for circuit refinement and neuron–glia communication [48]. For instance, early-life inflammation enhances microglial engulfment of glutamatergic spines in the anterior cingulate cortex, prolonging neuronal stress refractoriness and delaying depressive symptom onset [49,50]. Aberrant synaptic pruning and deficient remyelination are thus key pathological features in depression [51]. These structural changes underlie the aberrant connectivity observed in depression. The causal role of neuroinflammation is further supported by interventional evidence. Suppressing microglial-mediated inflammation can effectively ameliorate depressive-like behaviors, further supporting the theory that neuroinflammation contributes to the pathogenesis of depression [52]. Given their multifaceted impact on neural structure and function, microglia present compelling targets for next-generation antidepressant interventions.

## 3. Metabolic Reprogramming of Microglia

Microglial phenotypic changes are central to depression pathogenesis and deeply coupled to their metabolic activity. The metabolic state of microglia encompasses the integration of their major metabolic biochemical pathways, including glycolysis, oxidative phosphorylation, lipid synthesis and oxidation, amino acid cycling, and mitochondrial function. This integrated state dictates the availability of energy supply, biosynthetic building blocks, and signaling metabolites. This metabolic state is not static or unidirectional; rather, it serves as a dynamic driver of cellular phenotype and function. It undergoes reprogramming in response to stressors, inflammatory signals, and the local microenvironment during disease progression.

**Figure 1:**
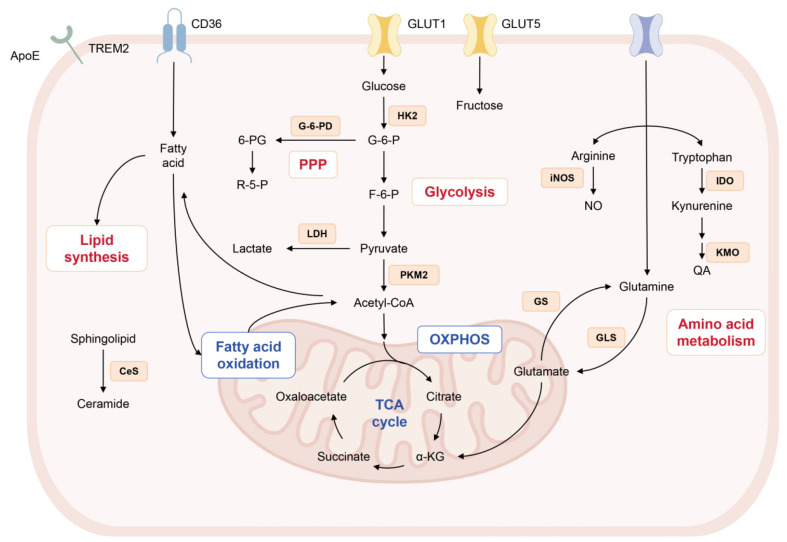
An overview of metabolic reprogramming in microglia in depression. In depression, microglia shift toward a pro-inflammatory phenotype accompanied by extensive metabolic reprogramming. A hallmark of this transition is enhanced glycolysis driven by upregulation of GLUT1, HK2, and PKM2 coupled with impaired OXPHOS, elevated lactate production, and increased flux through the PPP. Lipid metabolism is skewed toward accumulation, with reduced FAO and elevated ceramides that exacerbate membrane dysfunction and inflammation. Disruption of amino acid metabolism includes preferential metabolism of arginine via iNOS to generate nitric oxide, interconversion of glutamate and glutamine and shunting of tryptophan into the kynurenine pathway via IDO and KMO, leading to the neurotoxic metabolite quinolinic acid. Together, these interconnected metabolic alterations reinforce microglial inflammatory activation and drive the progression of depression pathology.

In recent years, immunometabolism has arisen as a major frontier in neurodegenerative disease research [53]. The brain demonstrates high energy demands, abundant lipid content, active mitochondrial metabolism, and elevated reactive oxygen species production, rendering it especially vulnerable to oxidative stress and inflammatory injury [54]. In depression, alterations in the metabolic state of microglia may represent a fundamental mechanism underlying their shift from a homeostatic surveillance phenotype to a dysfunctional pro-inflammatory or neurotoxic phenotype. Early stress stimuli may induce transient metabolic adaptations, whereas chronic stress can trap microglia in a sustained dysfunctional state. Neuroinflammatory signaling triggers extensive immunometabolic reprogramming in microglia. Multiple metabolic pathways respond in a coordinated, integrated manner, characterized by a shift in energy metabolism from efficient oxidative phosphorylation to rapid glycolysis, accompanied by disruption of lipid metabolic homeostasis and redirection of amino acid pathways. This reprogramming serves a dual purpose: on the one hand, it provides the energy and biosynthetic precursors required for the pro-inflammatory activation and phagocytic functions of microglia; on the other hand, metabolic intermediates generated in the process—such as lactate, succinate, and reactive oxygen species—also act as signaling molecules and precursors, participating in epigenetic and post-translational modifications. Together, they regulate the neural environment modulated by microglia (Figure 1). These metabolic alterations subsequently contribute to the pathological progression of depression by modulating the release of inflammatory cytokines, synaptic plasticity, and neurotransmission.

### 3.1. Glucose Metabolism

Glucose stands as a primary metabolic substrate in the brain, which depends heavily on it for energy production. Previous research has established that impaired cerebral glucose metabolism contributes to the pathogenesis of depression [55]. Patients with MDD show elevated cortical levels of glucose and lactate, which correlate with clinical severity [56]. Stable isotope-resolved metabolomics in a chronic unpredictable mild stress (CUMS) rat model revealed suppressed tricarboxylic acid (TCA) cycle flux, enhanced glycolysis, and activated gluconeogenesis, reflecting an accelerated yet inefficient glucose metabolic phenotype as an adaptive response to stress [57,58]. An FDG-PET study further demonstrated that microglia exhibit greater glucose uptake than neurons or astrocytes, underscoring their strong metabolic dependence on glucose and the significance of metabolic reprogramming in microglial phenotypic remodeling [59]. Together, these findings suggest that cerebral glucose metabolism may contribute to depressive pathogenesis by reshaping microglial inflammatory phenotypes [60].

#### 3.1.1. Glycolysis and Oxidative Phosphorylation

Glucose is primarily catabolized through glycolysis and OXPHOS. A growing body of evidence suggests that microglia can undergo metabolic reprogramming during inflammatory activation, shifting energy reliance from OXPHOS toward glycolysis as the dominant ATP-producing route accompanied by heightened glucose uptake and lactate fermentation [61,62]. A glycolytic shift now represents a hallmark of pro-inflammatory microglia, whereas anti-inflammatory microglia preferentially employ OXPHOS, which supports a more stable and efficient energy supply [63,64]. Pharmacological inhibition of glycolysis with 2-DG has been shown to suppress chronic stress-induced microglial activation and depressive-like behavior, confirming that metabolic reprogramming is essential for microglial phenotypic adaptation and depression pathogenesis [65,66]. An alternative perspective posits that microglia may enter a chronic tolerant state due to energy metabolism deficits, wherein both glycolysis and OXPHOS are suppressed, ultimately dampening immune responses such as cytokine release and phagocytic capacity [67]. This metabolic plasticity enables microglia to dynamically adapt their functional phenotypes under varying pathophysiological conditions.

This metabolic plasticity likely enables microglia to dynamically reallocate energy resources according to environmental cues and functional requirements. Glycolysis supports rapid ATP generation and glucose utilization, favoring swift responses during pro-inflammatory activation, such as inflammasome assembly and cytokine production. In contrast, surveillance-state and anti-inflammatory microglia rely on OXPHOS, which provides sustained, efficient ATP output for immune monitoring and tissue repair while minimizing energetic waste. Under chronic stress, however, this metabolic equilibrium becomes dysregulated. Excessive glycolysis may lead to bioenergetic insufficiency and impaired phagocytosis, while suppressed OXPHOS promotes mitochondrial fragmentation, ROS accumulation, and mitochondrial DNA release, collectively exacerbating neuroinflammation and neuronal damage [68,69,70]. Critical outstanding questions include how environmental signals initiate microglial metabolic reprogramming, and at what point adaptive metabolic shifts transition to maladaptive pathology. One emerging mechanism involves non-canonical functions of metabolic enzymes and intermediates involved in signaling cascades, post-transcriptional regulation and post-translational modifications, which may accelerate microglia into a pro-inflammatory state.

The molecular mechanisms governing metabolic switching in microglia remain incompletely defined; however, increased glucose uptake and upregulation of key glycolytic enzymes clearly contribute [71]. Glucose transporters (GLUTs) mediate transmembrane sugar uptake, with microglia expressing several isoforms. Among these, GLUT1 is the most abundant and serves as the primary route for glucose import, playing a critical role in promoting glycolysis [72]. Knockdown of GLUT1 attenuates LPS-induced microglial activation [65]. In CUMS model mice, hippocampal microglia exhibit elevated GLUT1, and its specific knockdown alleviates stress-induced spatial learning and memory deficits [73]. GLUT5, a fructose transporter, also participates. Early-life high-fructose intake impairs microglial phagocytosis and increases adolescent anxiety susceptibility, implicating GLUT5 in emotional regulation [74]. These findings underscore the importance of glucose transporter-mediated metabolic reprogramming in shaping microglial function and depression-related pathology.

Hexokinase 2 (HK2) catalyzes the first committed step of glucose metabolism in microglia, phosphorylating glucose to generate glucose-6-phosphate (G6P), and is critical for both glycolysis and mitochondrial respiration [75]. HK2 is significantly upregulated in pro-inflammatory microglia, increasing glycolytic flux; however, HK2 deficiency concurrently impairs both glycolysis and mitochondrial function, leading to energy deficit, elevated reactive oxygen species, and exacerbated inflammatory responses [76]. Moreover, HK2 inhibition upregulated lipoprotein lipase and enhanced lipid metabolism to increase ATP production, thereby promoting microglial phagocytosis and reducing amyloid-β deposition. These effects were reversed by supplemental G6P [77]. TSPO deficiency impairs mitochondrial respiration, leading to increased recruitment of HK2 to mitochondria, a shift toward glycolysis, and reduced microglial phagocytosis [78]. Emerging evidence also highlights the non-metabolic roles of HK2 in regulating cellular functions [79]. HK2 can inhibit autophagic flux by reducing the association of autophagosomes with lysosomes via decreased lysosomal-associated membrane protein abundance [80].

Pyruvate Kinase M2 (PKM2) catalyzes the final step of glycolysis, generating ATP and pyruvate [81]. Its activity is conformation-dependent: the tetrameric form exhibits high catalytic activity in the presence of fructose-1,6-bisphosphate, whereas the dimeric form translocates to the nucleus and regulates diverse cellular functions [82]. In microglia, LPS stimulation upregulates PKM2 and promotes dimer formation and nuclear translocation. Nuclear dimeric PKM2 interacts with the transcription factor ATF2, triggering inflammasome activation and pyroptosis [83]. PKM2 ablation attenuates microglial migration [84], and dimeric PKM2 can also act as a protein kinase, phosphorylating STAT3 to enhance pro-inflammatory cytokine secretion [85]. Pharmacological activation of PKM2 and glycolysis alleviated depression-like behaviors in a zebrafish model exposed to artificial light at night [86]. In contrast, intranasal PKM2 administration in a rat model of post-stroke depression improved oligodendrogenesis, reduced neuroinflammation and oxidative stress, and rescued depressive behaviors [87], suggesting context-dependent anti-inflammatory properties. These divergent findings highlight the complex, pleiotropic nature of PKM2 and support its potential as a therapeutic target in depression, pending further mechanistic clarification.

Metabolite accumulation during metabolic reprogramming can profoundly reshape cellular function. Patients with psychiatric disorders exhibit decreased brain pH, likely reflecting metabolic alterations and the buildup of acidic metabolites such as lactate [88]. Lactate, generated from pyruvate reduction, plays essential roles in nervous system physiology. Insufficient brain lactate impairs BDNF expression, synaptic plasticity, and neurogenesis [89]. Conversely, excessive lactate accumulation driven by metabolic, epigenetic, and post-translational mechanisms promotes microglial activation, with microglial lactate levels positively correlating with neuroinflammation severity [90]. While lactate can serve as an alternative energy substrate [11], it also induces pan-histone H4K12 lactylation, activating transcriptional programs that enhance glycolysis, mitochondrial oxidative stress, and NLRP3 inflammasome-associated autophagy, collectively driving microglial hyperactivation and dysfunction [91,92,93]. These findings position lactate not only as a metabolic intermediate but also as a signaling molecule that reinforces microglial inflammatory states in depression.

#### 3.1.2. Pentose Phosphate Pathway (PPP)

G6P, produced by hexokinase-mediated phosphorylation of glucose, can be diverted into the PPP to generate ribose-5-phosphate and nicotinamide adenine dinucleotide phosphate hydrogen (NADPH). This pathway primarily supports nucleotide biosynthesis and redox balance, contributing minimally to ATP production [94]. As a key electron donor, NADPH sustains fatty acid and steroid synthesis, aids DNA repair, fuels antioxidant systems, and acts as a substrate for NADPH oxidase (NOX2) in reactive oxygen species generation [95,96]. Its dual role in both antioxidative defense and pro-inflammatory oxidative burst positions makes NADPH a crucial metabolic node in microglial immune responses.

Emerging evidence points to dysregulation of the PPP in mood and psychiatric disorders, where the activity of its rate-limiting enzyme, glucose-6-phosphate dehydrogenase (G6PD), correlates with mitochondrial dysfunction, oxidative stress, and cerebral pH in brain tissue [97]. In microglia, activation via LPS and interferon shifts metabolism toward glycolysis and the PPP [98], an enhancement that may serve as an adaptive mechanism to counter oxidative stress and meet biosynthetic needs during proliferation [71,99]. G6PD, a highly expressed brain enzyme, catalyzes the first step of the PPP. Under chronic LPS-induced neuroinflammation, G6PD mRNA expression rises, and microglial activation coincides with PPP upregulation and NADPH accumulation. Increased NADPH then stimulates NOX2 and inducible nitric oxide synthase (iNOS), driving excessive ROS production that worsens oxidative stress and inflammation [100,101]. Pharmacological inhibition of G6PD attenuates LPS-triggered microglial activation and neurodegeneration. Nevertheless, how the PPP and G6PD are altered in microglia within depression models remains poorly explored.

### 3.2. Lipid Metabolism

**Figure 2:**
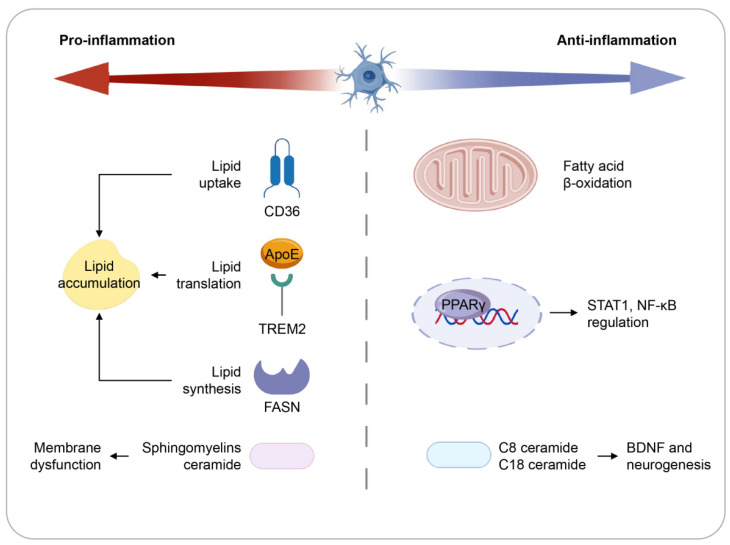
The lipid metabolic alternation in both pro- and anti-inflammatory microglia. Microglia display phenotype-specific lipid metabolic signatures that critically influence their inflammatory functions. Pro-inflammatory microglia enhance lipid uptake through CD36 and promote lipid transport via TREM2 and ApoE, while concurrently upregulating fatty acid synthesis collectively driving intracellular lipid accumulation that supplies precursors for inflammatory mediators and amplifies pro-inflammatory responses. In contrast, anti-inflammatory microglia preferentially engage in fatty acid oxidation, with PPARγ activation suppressing inflammation through inhibition of STAT1 and NF-κB signaling. Sphingomyelin metabolism is elevated in pro-inflammatory states, leading to increased ceramide production; however, certain ceramide species such as C8 and C18 exhibit context-dependent anti-inflammatory effects, underscoring the nuanced role of sphingolipids in microglial polarization.

Lipids, amphipathic and water-insoluble, act as energy sources, signaling molecules, and structural membrane components. The nervous system displays a high lipid content relative to other tissues [102]. Depression onset is associated with changes in diverse lipid species [103]. Long-term high-fat diet (HFD) intake induces anxiety- and depression-like behaviors and cognitive impairment, accompanied by lipid accumulation in microglia, leading to their hyperactivation or reduced density across brain regions and promoting aberrant neuronal remodeling [104]. In contrast, short-term lipid interventions show that microglia metabolize harmful fatty acids and release protective factors, enhancing learning and memory [105]. Microglia thus utilize unique lipid metabolic mechanisms to critically regulate emotional and cognitive functions (Figure 2).

#### 3.2.1. Lipid Accumulation

Lipid droplet (LD) accumulation in aged microglia reflects a functionally impaired state, defining a subtype termed lipid-droplet-accumulating microglia that exhibits impaired phagocytosis, elevated ROS, and heightened inflammatory activation [106]. LPS stimulation increases LD accumulation within microglia [107]. HFD-induced intracellular lipid deposition and HIF-1α upregulation trigger microglial hyperactivation, excessive phagocytosis of ventral hippocampal neurons and myelin, and subsequent depressive-like behaviors and neural dysfunction [108]. Although direct ultrastructural evidence for LD formation in microglia of depressed patients remains limited, substantial studies indicate a significant disruption in lipid metabolic homeostasis, with pathological features aligning with lipid dysregulation. Investigating lipid metabolic reprogramming in microglia in depression, particularly the imbalance between enhanced lipid accumulation and impaired catabolism, offers a fresh perspective for understanding the neuroimmune-inflammatory mechanisms underlying the disorder.

Lipid metabolism critically regulates the production of inflammatory cytokines and chemokines. Triglycerides (TGs), neutral lipids that store fatty acids, constitute the major component of LDs. Modulation of TG metabolism via diacylglycerol acyltransferase activity influences microglial responses to internal or external stressors, thereby affecting inflammatory signaling and disease-associated functions [109]. Hydrolysis of neutral lipids releases fatty acid precursors for inflammatory mediators such as prostaglandins, amplifying inflammatory responses [110]. Lipid accumulation in microglia is closely linked to enhanced lipid uptake, with the class B scavenger receptor CD36 serving as a key regulator. Upon lipid internalization, activation of the nuclear factor erythroid 2-related factor 2 (NRF2) pathway promotes CD36 expression through a positive feedback mechanism [111,112]. CD36 binds diverse lipid ligands, including long-chain fatty acids and oxidized low-density lipoprotein, and facilitates fatty acid uptake via receptor-mediated endocytosis [113]. The pro-inflammatory cytokine IL-21 upregulates CD36 and promotes lipid accumulation in microglia [114], while CD36 deletion suppresses pro-inflammatory polarization by downregulating the Traf5-MAPK pathway [115]. Additionally, CD36 interacts with Toll-like receptor (TLR) signaling; the anti-CD36 antibody inhibits LPS-induced TLR2/3 expression [116] and modulates caspase-1 activity to regulate IL-1β production [117].

Enhanced lipid uptake is closely associated with an elevated capacity for intracellular lipid transport. Apolipoprotein E (apoE), a lipid-binding protein encoded by a major genetic risk factor for AD, has recently been strongly implicated in the pathophysiology of anxiety and depressive disorders [118,119,120]. ApoE facilitates the intercellular and interorgan transport of cholesterol and phospholipids, playing a pivotal role in the regulation of nervous system lipid metabolism [121]. In microglia and neurons, the ApoE4 allele promotes LD accumulation, thereby enhancing microglial phagocytosis of synapses [122]. Studies employing CUMS in apoE-targeted replacement mice reveal that apoE expression can induce depression-like behaviors and cognitive deficits [123]. At the cellular level, microglial apoE3 enhances antigen presentation and interferon signaling, whereas apoE4 promotes stress responses, induces LD formation, and disrupts neuron–microglia communication [124,125]. Notably, under CUMS conditions, ApoE-knockout mice exhibit exacerbated depression-like behaviors, suggesting a complex, context-dependent regulatory function of apoE in depression pathogenesis [126]. ApoE-driven LD biogenesis in microglia involves the triggering receptor expressed on myeloid cells 2 (TREM2) [127]. As a transmembrane regulator of lipid homeostasis, TREM2 modulates cholesterol uptake and metabolic accumulation in microglia. TREM2 deficiency exacerbates lipid droplet accumulation and modulates neuroinflammation via the TGF-β1/Smad2/3 signaling axis [128]. Corticosterone-induced stress downregulates TREM2 expression in microglia and regulates inflammatory cytokine production through the JAK/STAT3 pathway [129]. Suppression of TREM2 in microglia is sufficient to induce depression-like behaviors in mice. Furthermore, TREM2 expression is transcriptionally regulated by NRF2 and ultimately influences the BDNF-TrkB signaling pathway [130]. TREM2 also functions as a critical immune receptor linking peripheral inflammation to microglial dysfunction and the manifestation of depression-like symptoms [131].

Consistent with a lipid-accumulating phenotype, immune cell activation is associated with reduced expression of lipolytic enzymes [132]. Paradoxically, however, specific knockout of adipose triglyceride lipase (ATGL) in microglia attenuates neuroinflammatory factors such as IL-6 and IL-1β and reduces anxiety-like behaviors [133]. This effect may stem from decreased availability of free fatty acids, such as arachidonic acid, which serve as substrates for inflammatory mediators derived from triglyceride lipolysis. Lipoprotein lipase (LPL) hydrolyzes triglyceride-rich lipoproteins to release free fatty acids; its knockout leads to marked lipid droplet accumulation in microglia, impaired cholesterol efflux, and redirection of lipids toward eicosanoid and polyunsaturated fatty acid synthesis, potentially mediated by reduced peroxisome proliferator-activated receptor gamma (PPARγ) expression and fatty acid uptake [134]. PPARγ, a nuclear transcription factor involved in metabolic gene regulation, exerts anti-inflammatory and neuroprotective effects [135]. In microglia of mice subjected to CMS, PPARγ expression decreases alongside increased phosphorylation, favoring a pro-inflammatory phenotype [136]. FOXO3a downregulation promotes PPARγ expression, which suppresses alternative microglial activation and alleviates LPS-induced depression-like behaviors in mice [137]. PPARγ also modulates NLRP3 inflammasome activation via STAT1 signaling [138] and inhibits NF-κB to regulate pro- and anti-inflammatory cytokine secretion [139]. Through these mechanisms, PPARγ serves as a critical node linking lipid metabolism to neuroinflammation and the progression of depression.

Recent studies suggest that lysosomal dysfunction caused by lipofuscin deposition may play a key role in impairing microglial function and promoting oxidative stress in aging and neurodegenerative diseases [140]. Lipofuscin is an autofluorescent, electron-dense pigment composed of heavily oxidized proteins, lipids, and metals. Due to their constant phagocytosis and remodeling of myelin, microglia may be among the first cells in the central nervous system to accumulate lipofuscin [141]. Lipofuscin deposition impairs lysosomal acidification and enzymatic activity, leading to the buildup of metabolic waste in microglia and exacerbating oxidative stress and inflammation. In AD models, reacidification of microglial lysosomes can rescue toxic protein aggregation and neuroinflammation, highlighting the importance of functional lysosomes in maintaining microglial homeostasis and slowing neurodegeneration [142]. Although research on lipofuscin in the context of chronic stress or mood disorders is limited, its close link to oxidative stress and disrupted lipid metabolism makes it a potential research focus for understanding neuropathology and microglial dysfunction in long-term or treatment-resistant depression.

#### 3.2.2. Fatty Acid Synthesis and Oxidation

Fatty acids are essential energy substrates that are catabolized through fatty acid oxidation (FAO) to generate ATP, with β-oxidation representing the major pathway for mitochondrial degradation into acetyl-CoA and subsequent entry into the TCA cycle. Very long-chain fatty acids (VLCFAs) are initially metabolized in peroxisomes [143]. MDD is characterized by functional deficits in fatty acid metabolism, as evidenced by reduced plasma levels of medium- and long-chain acylcarnitines in patients, which increase following treatment, suggesting impaired fatty acid β-oxidation during the disease state [144]. Postmortem analyses reveal downregulated expression of key genes involved in fatty acid synthesis in the prefrontal cortex (PFC), indicating a disruption in long-chain polyunsaturated fatty acid production [145]. In peripheral macrophages, FAO is modulated by inflammatory mediators [146]. Microglia, which express multiple FAO-associated enzymes such as acyl-CoA synthetase and LPL, demonstrate a capacity for utilizing FAO [147,148]. Furthermore, alterations in fatty acid synthesis or oxidative metabolism occur during microglial activation.

Activation of primary mouse microglia with IL-1β/IFN-γ induces pronounced downregulation of key enzymes involved in mitochondrial and peroxisomal fatty acid oxidation along with reduced expression of fatty acid synthase (Fasn) and diminished activity of the fatty acid synthesis pathway. In contrast, IL-4-polarized anti-inflammatory microglia exhibit enhanced fatty acid oxidation activity [149]. Impairment of FAO, particularly dysfunctional peroxisomal β-oxidation, leads to intracellular accumulation of VLCFAs, which disrupts glycerophospholipid composition in immune cell membranes and impairs cytoskeletal remodeling during activation, ultimately causing phagocytic deficits and altered cytokine secretion [150]. In BV2 microglia with genetic defects in peroxisome-related genes, transcriptomic reprogramming alters lipid metabolism, autophagy, and immune responses, accompanied by redistributed cholesterol and lipid droplets, reflecting a DAM phenotype [151,152]. Upon LPS stimulation, these cells display amplified inflammatory activation, including enhanced inflammasome activity, cytokine release, antigen presentation, phagocytosis, and T cell co-stimulatory responses [153]. Carnitine palmitoyltransferase 1A (CPT1A), which mediates mitochondrial fatty acid import, plays a central role in microglial lipid metabolism. Astrocyte-derived IL-3 upregulates CPT1A expression in microglia, thereby improving lipid metabolism, increasing membrane glycerophospholipid levels, and reducing pro-inflammatory activation and associated neuronal damage [154]. Notably, long-chain acyl-coenzyme A synthase 4 (ACSL4) expression is upregulated in LPS-stimulated microglia, leading to VGLL4 downregulation and potentiation of NF-κB signaling. Knockdown of ACSL4 modulates inflammation by altering cellular lipid composition, including phospholipids, sphingolipids, and fatty acids [155]. Collectively, activated microglia shift toward reduced fatty acid oxidation consistent with downregulated mitochondrial TCA cycle activity, while favoring fatty acid synthesis and supporting a lipid-accumulating phenotype.

#### 3.2.3. Sphingolipids and Ceramides

Sphingolipids are a class of lipids defined by a sphingosine backbone, comprising sphingomyelins, ceramides, and glycosphingolipids. They function as vital structural elements in cell membranes and contribute to membrane fluidity [156]. Ceramides serve as key bioactive lipids that mediate signaling pathways involved in apoptosis, senescence, and differentiation. The accumulation of ceramides and associated lipotoxic inflammation has been documented in various neurodegenerative diseases [157].

Dysregulation of sphingolipid metabolism and elevated ceramide levels are strongly linked to MDD [158]. Membrane lipid analysis profiling of adolescent MDD patients revealed increased membrane levels of cholesterol, sphingol, and ceramide, alongside reduced polyunsaturated fatty acids, which may decrease membrane fluidity and promote inflammation [159]. Direct infusion of C20-ceramide into the rat ventral hippocampus induced anhedonia-like behavior, supporting a role for ceramide not only as a biomarker but also as an active contributor to depressive pathology, although local ceramide administration did not trigger microglial activation [160]. Synergistic exposure to palmitic acid and LPS promotes de novo ceramide synthesis and sphingomyelin hydrolysis, amplifying pro-inflammatory cytokine expression, though the mechanistic link between stimulation and ceramide accumulation remains unclear [161]. One proposed pathway involves ceramide-driven activation of the NLRP3 inflammasome, where heightened sphingolipid turnover and elevated ceramide synthase 5 (CerS5) expression trigger NLRP3 signaling, promoting microglial pyroptosis and neuronal injury [162,163]. Additionally, reduced phosphatidylserine and sphingomyelin in microglia impair membrane assembly of complement C3, disrupting synaptic pruning signals and contributing to depression-like and bipolar disorder-like behaviors [164]. Conversely, certain ceramide subspecies may exert protective effects. Exercise upregulates ceramide synthase 1 (CerS1) in microglia, boosting C18-ceramide production and alleviating depressive-like behaviors, while CerS1 overexpression suppresses neuroinflammation induced by chronic stress or LPS [165]. Similarly, C8-ceramide modulates microglial BDNF to support cognitive function [166]. These data indicate that ceramides regulate microglial activity and neural circuits in a context- and acyl chain-specific manner, rather than through uniform pro-inflammatory effects.

### 3.3. Amini Acid Metabolism

Amino acids, as one of the three major nutrient classes alongside carbohydrates and lipids, play a critical role in microglial metabolism. Studies demonstrate that under conditions of glucose insufficiency, amino acids can serve as alternative energy sources to sustain basic microglial functions, highlighting the metabolic plasticity and environmental adaptability of these cells [167]. Beyond their role as metabolic substrates, amino acids are essential for the synthesis of biologically active proteins and function as key signaling molecules that directly regulate cellular activities. These mechanisms collectively underscore the central importance of amino acids in mediating microglial functions such as immunomodulation, phagocytosis, and inflammatory responses.

#### 3.3.1. Arginine Metabolism

Arginine, a semi-essential amino acid, is synthesized from citrulline in the kidneys but requires dietary supplementation under high-demand conditions [168]. In myeloid cells, arginine metabolism proceeds through two major pathways: one involves catalysis by arginase 1 or 2 (ARG1/ARG2), processes essential for cell proliferation and tissue repair. The other pathway, induced during pro-inflammatory activation, is mediated by iNOS, which metabolizes arginine to citrulline and nitric oxide (NO) [169]. In the central nervous system, NO functions as a pleiotropic neurotransmitter with both excitatory and inhibitory roles, and its dysregulation contributes to psychiatric disorders such as depression via mechanisms involving HPA axis dysfunction, serotonin signaling alterations, and neuroinflammation [170].

Of note, these two distinct metabolic pathways critically modulate microglial activation states. iNOS is a well-established marker of pro-inflammatory microglial activation, whereas ARG1 expression is associated with the resolution of neuroinflammation and neuroprotection [18,20]. Under homeostatic conditions, microglia express minimal iNOS; however, its mRNA is significantly upregulated in the cortex and hippocampus in CUMS and CSDS mouse models of depression [136,171]. LPS-induced depression models further confirm that increased microglial iNOS promotes inflammatory factor release and exacerbates neuroinflammation [172]. While iNOS ablation enhances antidepressant-like effects in chronically stressed mice, it also reduces neurogenesis and induces anxiety-like behavior, suggesting a dual role for iNOS beyond inflammation, potentially involving neural repair processes [173]. Conversely, a distinct population of ARG1^+^ microglia resides in the basal forebrain and ventral striatum of healthy mice, and conditional knockout of ARG1 impairs neural plasticity and cognition [174]. In CUMS models, the number of Arg1^+^ microglia decreases. Pharmacologically inducing an Arg1^+^ phenotype in microglia ameliorates chronic stress-induced inflammation and synaptic damage, exerting antidepressant effects [175,176]. Similarly, in an atopic dermatitis model, loss of Arg1^+^ microglia reduces hippocampal BDNF and pCREB, triggering depression-like behaviors, further underscoring their protective role in mood regulation [177]. Collectively, the balance between iNOS-driven and ARG1-driven arginine metabolism in microglia represents a critical regulatory node in neuroinflammation and depression pathophysiology.

#### 3.3.2. Glutamine/Glutamate Metabolism

Glutamate, the principal excitatory neurotransmitter in the central nervous system, regulates brain connectivity by modulating the excitatory–inhibitory equilibrium, and its dysregulation contributes to the pathogenesis of affective disorders [178]. Glutamine, the amide derivative of glutamate, serves as an important alternative metabolic substrate under glucose-deficient conditions, enabling microglia to sustain process motility and sensory functions [167]. The interconversion of glutamine and glutamate is catalyzed by glutaminase (GLS) and glutamine synthetase (GS). Glutamate can be further metabolized by glutamate dehydrogenase (GLUD) or transaminases to yield α-ketoglutarate (α-KG), which replenishes the TCA cycle [179,180]. This integration highlights the role of glutamate–glutamine cycling not only in neurotransmission but also in microglial bioenergetics.

Emerging evidence indicates that upregulated GLS activity and the resulting glutamate excess contribute to excitotoxicity and sustained immune activation in the central nervous system [181,182]. Altered GLS expression may represent a key mechanism underlying microglial phenotypic switching and inflammatory regulation [183]. Elevated GLS1 mRNA levels and enrichment of neuroinflammatory pathways have been observed in the prefrontal cortex of depressed individuals and in microglia from CRS model mice. Specific knockout of GLS1 in microglia or its pharmacological inhibition alleviates neuroinflammation, depression-like behaviors, and aberrant microglial synaptic pruning [184,185]. In vitro, LPS stimulation increases GLS expression in microglia, and GLS overexpression promotes a pro-inflammatory phenotypic shift [186]. As activated immune cells often favor glycolysis, glutamine metabolism may serve as an anaplerotic substrate for the TCA cycle, supporting oxidative phosphorylation to meet energy demands and provide carbon/nitrogen for biosynthetic precursors and reducing equivalents [187,188]. These findings collectively position glutaminolysis at the intersection of microglial immunometabolism and excitotoxicity, highlighting its therapeutic potential in mood disorders. Multiple studies link glutamine metabolism to NLRP3 inflammasome activation. NLRP3 activation is accompanied by enhanced glutamine utilization and α-KG flux into the TCA cycle, along with increased production of glutamine-derived amino acids such as aspartate. Inhibition of GLS promotes mitophagy, reduces intracellular ROS, and consequently suppresses NLRP3 inflammasome activation and IL-1β release [189]. In contrast, another study indicates that NLRP3 inhibition in microglia increases glutamine hydrolysis and α-KG levels, enhancing phagocytic function through histone acetylation of genes including Slc1a3, Cd68, and Cd300lf [190]. Meanwhile, pro-inflammatory stimuli significantly upregulate GS in microglia [191,192]. GS inhibition exacerbates the inflammatory response in LPS-activated microglia [193]. While GS is classically associated with astrocytes for glutamate clearance, microglia under inflammatory conditions upregulate this enzyme. Unstimulated microglia exhibit minimal capacity to convert glutamate to glutamine [191], though expression of the glutamate transporter GLT-1 in adult rat facial nucleus-derived microglia suggests a latent ability for glutamate uptake [194]. Together, these findings indicate that dysfunctional microglia in depression are characterized not by a simple change in glutamine or glutamate levels, but by an imbalance in the glutamine/glutamate ratio. Disrupted glutamine metabolism during microglial activation and neuroinflammation likely represents a key contributor to microglial dysfunction in the pathophysiology of depression.

#### 3.3.3. Tryptophan/Kynurenine Metabolism

Tryptophan, a precursor for serotonin synthesis, plays a critical role in depression pathology. Tryptophan is diverted into the kynurenine pathway via metabolism by IDO, yielding kynurenine. Within microglia, kynurenine is further metabolized by kynurenine 3-monooxygenase (KMO) into 3-hydroxykynurenine (3-HK) and quinolinic acid (QA) [195]. QA serves as a precursor for nicotinamide adenine dinucleotide (NAD^+^) biosynthesis but also induces mitochondrial dysfunction and ROS production. It functions as a neurotoxin by activating N-methyl-D-aspartic acid receptors, leading to glutamatergic dysregulation and calcium-mediated excitotoxicity [196,197,198]. Through its bioactive metabolites, the kynurenine pathway integrates inflammatory signaling, neuroactive regulation, and oxidative stress, thereby contributing centrally to the pathophysiology of depression [47].

KP metabolizes approximately 95% of tryptophan and is centrally implicated in neuroinflammatory processes and the pathogenesis of depression through its dysregulation. In LPS-challenged mice, depression-like behaviors are associated with significant accumulation of 3-HK in the hippocampus [199]. Studies using CUMS models link IDO and KMO activity to depressive-like behaviors, alongside alterations in quinolinic acid and kynurenic acid levels [200,201,202]. KP metabolites may contribute to cognitive impairment in mood disorders by disrupting neurovascular communication via hemodynamic changes in the choroid plexus [203]. However, chronic stress-induced KP dysregulation may stem from peripheral kynurenine crossing the blood–brain barrier and activating microglia [204], raising questions about the primary cellular source of kynurenine. HFD disrupts tryptophan metabolism, increasing neurotoxic metabolites and reducing indole derivatives, thereby linking obesity to depression [205]. IDO, enriched in brain macrophages/microglia at parenchymal interfaces, regulates phagocytosis by modulating membrane dynamics [206]. Inflammation upregulates IDO and KMO in microglia [207], and pharmacological inhibition of these enzymes prevents LPS-induced depression-like behaviors [208,209]. Collectively, KP dysregulation represents a key mechanism bridging metabolic imbalance, neuroinflammation, and depressive pathology.

### 3.4. Mitochondrial Function and TCA Cycle

The TCA cycle functions as a central bioenergetic hub, integrating catabolic pathways of glucose, lipids, and amino acids. Under homeostatic conditions, microglia depend predominantly on mitochondrial TCA cycle activity for energy generation [210]. Accumulating evidence reveals mitochondrial dysfunction and disrupted energy metabolism in both individuals with depression and animal models, supporting a growing perspective that depression may be conceptualized as a mitochondrial-associated disorder [211,212].

Mitochondria, as double-membrane-bound organelles, possess an autonomous quality control system and undergo continuous cycles of fusion and fission, processes essential for maintaining functional homeostasis [213]. Mitochondrial fission is primarily regulated by dynamin-related protein 1 (DRP1), which facilitates outer membrane constriction [214], while fusion is mediated by mitofusins (MFN1 and MFN2) for outer membrane integration and optic atrophy 1 (OPA1) for inner membrane fusion and cristae formation [215]. Investigations into mitochondrial dynamics in depression models have revealed contrasting findings: CUMS elevates DRP1 levels, promoting fission and oxidative stress in the mouse brain [216], whereas CMS upregulates MFN, OPA1, and phosphorylated DRP1, indicating a shift toward fusion alongside enhanced biogenesis and mitophagy [217]. These disparities underscore that mitochondrial fusion and fission are dynamically balanced to support oxidative phosphorylation, and their dysregulation can impair metabolic function [218]. However, research specifically exploring mitochondrial dynamics in microglia within depressive contexts remains scarce. CMS increases mitochondrial mass in microglia, aligning with their activation [217]. LPS activation via TLR4 signaling induces mitochondrial fragmentation in microglia, consistent with a glycolytic shift in pro-inflammatory states [68]. This fragmentation compromises respiratory chain activity, elevates ROS production, and amplifies pro-inflammatory signaling through MAPK and NF-κB pathways [219]. Notably, inhibiting DRP1 dephosphorylation or overexpressing MFN1 and OPA1 attenuates LPS-induced inflammation in microglial models, highlighting the therapeutic potential of modulating mitochondrial dynamics to mitigate neuroinflammation [220,221].

The TCA cycle not only fuels bioenergetic pathways but also orchestrates cellular signaling and function through its intermediate metabolites. In inflammatory contexts, succinate accumulates in macrophages, stabilizing HIF-1α and promoting IL-1β production to exert pro-inflammatory effects [222]. Paradoxically, succinate may also suppress pro-inflammatory polarization in microglia by reducing mitochondrial fission and ROS generation [223]. Under chronic inflammatory or aging conditions, elevated succinyl-CoA availability promotes mitochondrial protein succinylation, while genetic deletion of the desuccinylase SIRT5 enhances glycolysis, lipid accumulation, and peroxidation [224]. Fumarate demonstrates anti-inflammatory activity via its derivative dimethyl fumarate, which engages the HCAR/Nrf2 pathway and produces antidepressant-like effects in model systems [225]. Similarly, inflammatory stimuli induce citrate accumulation, and inhibition of citrate synthase ameliorates microglial inflammation independently of metabolic reversal [226]. These findings underscore the TCA cycle’s central role in immunometabolic regulation and its potential as a target for modulating neuroinflammation.

**Figure 3:**
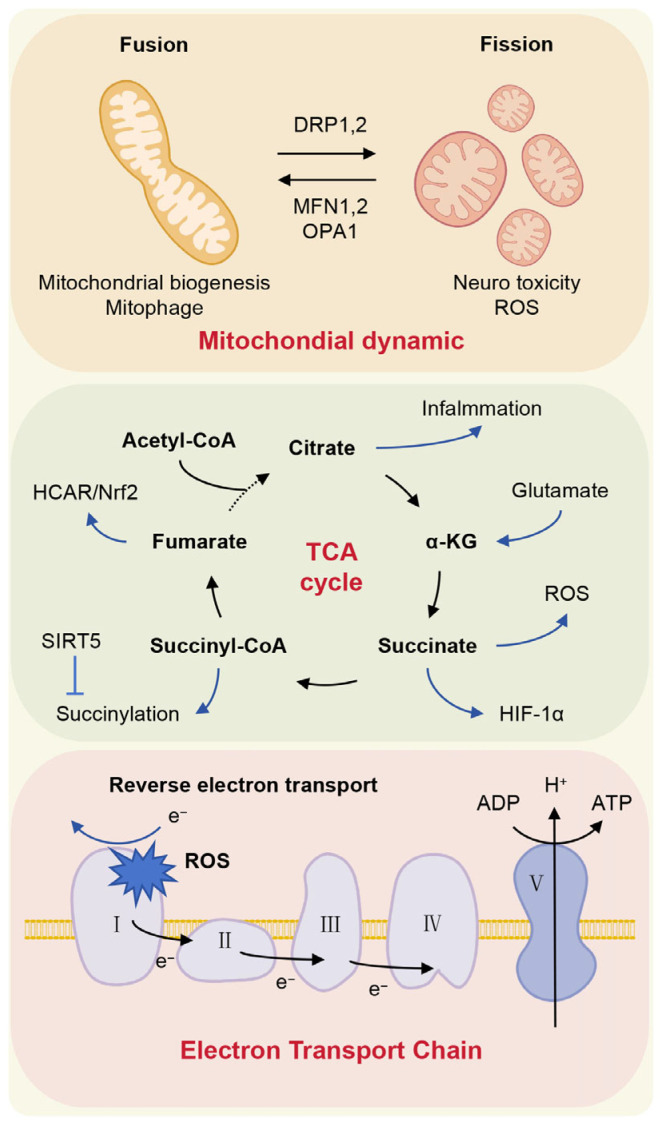
Mitochondrial Function in Microglial Metabolic Reprogramming. Mitochondria, as central metabolic hubs, undergo profound functional remodeling in microglia during phenotypic polarization. Pro-inflammatory activation reshapes mitochondrial dynamics through coordinated regulation of fission and fusion proteins including DRP1, MFN1, and OPA1, to alter organelle architecture and function. Key TCA cycle intermediates concurrently participate in inflammatory signaling: succinate stabilizes HIF-1α and amplifies ROS generation, while succinyl-CoA drives protein succinylation modifications. Fumarate activates the HCAR/Nrf2 anti-inflammatory pathway, and citrate accumulates under inflammatory stimulation. Furthermore, reverse electron transport at complex I exacerbates oxidative stress. Together, these mitochondrial processes orchestrate microglial metabolic reprogramming through tightly interconnected mechanisms.

In microglia undergoing chronic, low-grade inflammatory activation, mitochondrial complex I-driven reverse electron transport (RET) represents a key source of oxidative stress and neurotoxicity [227]. Paradoxically, RET-derived superoxide can also trigger a protective signaling cascade through UCP2 and AMPK activation, ultimately suppressing neuroinflammation [228], highlighting a context-dependent duality in RET-mediated signaling. Further underscoring mitochondrial dysregulation, LPS challenge impairs oxidative phosphorylation (OXPHOS) capacity without altering the protein levels of electron transport chain complexes [229]. Together, these findings position mitochondria as critical signaling hubs that integrate stress cues, bioenergetic status, redox balance, and immune output in microglia during depression. Disruption of mitochondrial homeostasis characterized by dysfunctional mitochondrial dynamics, imbalanced TCA cycle production and abnormal electron transport leads to maladaptive microglial responses and sustains neuroinflammation. A deeper understanding of microglia-specific mitochondrial pathways may therefore illuminate novel therapeutic strategies for depression and related neuropsychiatric disorders (Figure 3).

Microglial activation is underpinned by dynamic immunometabolic reprogramming, a process in which metabolic adaptations reciprocally dictate phenotypic polarization. This reprogramming supplies bioenergetic and biosynthetic support to bolster inflammatory responses and phagocytic capacity, while metabolic intermediates contribute to intercellular signaling, epigenetic regulation of inflammatory genes, and post-translational protein modifications [230]. Pro-inflammatory activation aligns with acute energy demands, characterized by enhanced glycolysis, lipid accumulation, and glutamine metabolism, whereas anti-inflammatory states preferentially utilize oxidative phosphorylation and fatty acid oxidation. Shifts in arginine and tryptophan metabolism primarily generate neuroactive metabolites for neural regulation rather than energy production. Furthermore, glucose, lipid, and amino acid metabolic pathways interact by sharing substrates such as acetyl coenzyme A, cross-regulating metabolic intermediates, and converging on mitochondria. In response to stress signals, they collectively regulate the overall metabolic state. Enhanced glycolysis influences flux through the pentose phosphate pathway, thereby altering antioxidant capacity. Lipid accumulation and impaired oxidation, in turn, feedback to inhibit mitochondrial function. Amino acid metabolism provides anaplerotic support for these pathways. Together, these interactions determine the phenotype and function of microglia. The metabolic reprogramming of microglia is not merely an adaptation to stress and inflammation; it may also serve as a critical link in depression, connecting an individual’s stress susceptibility and environmental pressure to neuroinflammation and brain plasticity.

However, translating these preclinical discoveries into clinical applications faces significant challenges. A primary obstacle is the lack of reliable, non-invasive, and specific indicators of microglial metabolic activity in vivo. Whether metabolic alterations observed in specific brain regions in models, including lactate accumulation, shifts in lipid profiles, and changes in specific neurotransmitter levels, can serve as stable and reliable peripheral biomarkers detectable in patient blood or cerebrospinal fluid still requires validation through large-scale clinical cohort studies. In neuroimaging, while [^18^F] FDG-PET has revealed aberrant cerebral glucose metabolism in depressed patients, with microglia contributing significantly to the signal [231], clinically available imaging tools currently lack the specificity to trace distinct metabolic pathways within microglia. Moving forward, developing novel PET tracers targeting key metabolic checkpoints, or leveraging advanced magnetic resonance spectroscopy techniques, will be pivotal for translating these fundamental discoveries into clinical tools for disease stratification and treatment monitoring.

## 4. Therapeutic Strategies Targeting Metabolic Pathways for Depression

Current first-line antidepressant therapies including selective serotonin reuptake inhibitors (SSRIs), serotonin–norepinephrine reuptake inhibitors (SNRIs), monoamine oxidase inhibitors, tricyclic antidepressants, and tetracyclic antidepressants are predicated on the monoaminergic neurotransmission dysfunction hypothesis, primarily acting to elevate synaptic levels of serotonin, norepinephrine, and/or dopamine [232]. Despite their widespread use, these agents are frequently limited by adverse effects. Growing evidence indicates that antidepressants influence cellular metabolism, offering fresh perspectives on their therapeutic efficacy and side effect profiles. Future research should prioritize investigating how these drugs modulate metabolic pathways and explore glial cell metabolism as a novel target for antidepressant development.

Although SSRIs and SNRIs have demonstrated anti-inflammatory effects in microglia [233,234,235], their direct impact on glial cell metabolism remains poorly characterized. Emerging evidence indicates that antidepressants can directly modulate mitochondrial function, though these effects appear highly context-dependent. An in vitro screen of multiple antidepressants in BV2 microglial cells revealed that bupropion, imipramine, trazodone, paroxetine, and venlafaxine elevated ROS and NO production while suppressing mitochondrial complex I and IV activity; paroxetine exhibited the strongest effect and additionally activated the inflammasome [236]. Studies on isolated mitochondria further showed that various antidepressants selectively inhibit complex I- or II-driven respiration, while uniformly suppressing complex IV activity [237,238,239]. Given the established role of mitochondrial dysfunction in MDD, such respiratory impairments may contribute to both the therapeutic and adverse effects of these drugs. Beyond mitochondria, antidepressants interact with diverse metabolic pathways: citalopram binds directly to GLUT1, inhibiting glucose uptake and glycolysis in hepatocellular carcinoma cells [240], while paroxetine enhances phosphofructokinase (PFK) activity, potentially altering glutamate and purine metabolism [241]. Several antidepressants also inhibit G6PD [242]. Conversely, not all metabolic effects are detrimental; in a CMS rat model, venlafaxine normalized stress-induced disruptions in glycolysis and biogenic amine metabolism, restoring physiological levels of glucose, lactate, and ATP [243]. These findings underscore that antidepressants exert multifaceted, often bidirectional, effects on cellular metabolism, highlighting the need to explore glial-specific metabolic mechanisms to fully understand their efficacy and side effect profiles.

Furthermore, targeting metabolic pathways represents an emerging therapeutic strategy for modulating neuroinflammation and microglial dysfunction. Capsaicin, a TRPV1 channel agonist known to alleviate neuropathic pain, has recently been shown to reverse microglial metabolic impairment. It enhances OXPHOS, reduces ROS production, and protects mitochondrial membrane potential via the mTOR–AKT–HIF1α axis [244]. Additionally, TRPV1 activation reverses ApoE4-induced lipid droplet accumulation in microglia, improving immune function and attenuating neuron loss and memory deficits [122]. Capsaicin also ameliorates LPS-induced depression-like behaviors in mice, elevates serotonin levels, and synergizes with amitriptyline to produce antidepressant effects [245,246]. Similarly, the flavonoid rutin exhibits anti-inflammatory, hypoglycemic, and neuroprotective properties. In microglia, sodium rutin restores OXPHOS capacity and reverses the LPS-induced glycolytic shift, thereby improving energy metabolism [247]. Its antidepressant actions involve PPARγ activation and IDO inhibition, underscoring a multi-target metabolic role [248,249]. PPARγ agonists such as rosiglitazone and pioglitazone promote cholesterol efflux and suppress lipid droplet accumulation in microglia, curbing inflammatory activation [134] and supporting remyelination [250]. Pioglitazone alleviates depression-like behaviors in CMS-exposed mice and induces a neuroprotective microglial phenotype [251], while rosiglitazone improves neurobehavioral deficits in an autoimmune model by inhibiting ACSL4 [252]. Together, these findings highlight metabolic reprogramming as a viable avenue for treating depression through glial-directed mechanisms. However, when evaluating the translational potential of treatment strategies based on animal models, it is important to note that species differences between animals and humans in the immune system, microglial gene expression profiles, and details of metabolic pathways may affect the ultimate efficacy of therapies targeting specific metabolic enzymes. Future research should conduct bidirectional validation by integrating mechanistic findings from animal models with neuroimaging, peripheral biomarkers, and neuropathological studies in depressed patients. Concurrently, longitudinal studies tracking changes in microglial metabolic states with the progression of depression, along with more rigorous and precisely designed randomized controlled trials, will be essential to extend and realize clinical translation.

## 5. Conclusions

The pathogenesis of depressive disorders emerges from complex interplays between individual vulnerability and environmental stressors, yet the biological underpinnings of such variability remain poorly defined, and reliable biomarkers for risk assessment or disease tracking are still lacking. Multi-omics approaches have begun to unveil the dynamic heterogeneity of microglia across physiological and pathological states. These cells contribute to depression through roles in neuroinflammation, neurotransmission, synaptic remodeling, and other neural processes. While this functional diversity complicates precise mechanistic attribution, it also unveils new therapeutic opportunities for depression and related neurodegenerative conditions. Critically, microglia display remarkable metabolic plasticity, allowing adaptation to microenvironmental shifts; metabolic imbalance may skew phenotypic polarization and amplify pathogenic functions. Moving forward, research must delineate the bidirectional relationship between depressive progression and microglial metabolic reprogramming, while integrating temporal dynamics of cellular states with clinical subtypes and disease stages for paving the way for precisely timed, pathology-aware intervention strategies.

Targeting glial cells represents a promising frontier in the development of novel antidepressant therapies, though further validation of their efficacy and safety is still needed. In contrast to conventional antidepressants that directly modulate neuronal neurotransmission, strategies focused on resolving neuroinflammation and restoring microglial metabolic homeostasis may present a superior safety profile by circumventing direct interference with neuronal signaling pathways. This approach could significantly broaden the therapeutic arsenal for treatment-resistant depression. Moreover, given the high comorbidity of depression with conditions such as cancer, traumatic injury, autoimmune disorders, and neurodegeneration, investigating how pharmacological interventions influence microglial metabolism and inflammatory responses may open avenues for personalized treatment strategies tailored to specific pathological contexts.

## Data Availability

No new data were created or analyzed in this study. Data sharing is not applicable to this article.

## Abbreviations

HPA	hypothalamic-pituitary-adrenal
CNS	central nervous system
ROS	reactive oxygen species
OXPHOS	oxidative phosphorylation
ATP	adenosine triphosphate
AD	Alzheimer’s disease
scRNA-seq	single-cell RNA sequencing
DAM	disease-associated microglia
dlPFC	dorsolateral prefrontal cortex
ACC	anterior cingulate cortex
MDD	major depressive disorder
TSPO	translocator protein
CUS	chronic unpredictable stress
CRS	chronic restraint stress
CSDS	chronic social defeat stress
LPS	lipopolysaccharide
BDNF	brain-derived neurotrophic factor
IDO	indoleamine 2,3-dioxygenase
CUMS	chronic unpredictable mild stress
TCA	tricarboxylic acid
GLUTs	glucose transporters
HK2	hexokinase 2
G6P	glucose-6-phosphate
PKM2	pyruvate kinase M2
PPP	pentose phosphate pathway
NADPH	nicotinamide adenine dinucleotide phosphate hydrogen
NOX2	NADPH oxidase
G6PD	glucose-6-phosphate dehydrogenase
iNOS	inducible nitric oxide synthase
HFD	high-fat diet
LD	lipid droplet
TGs	triglycerides
NRF2	nuclear factor erythroid 2-related factor 2
TLR	toll-like receptor
apoE	apolipoprotein E
TREM2	triggering receptor expressed on myeloid cells 2
ATGL	adipose triglyceride lipase
LPL	lipoprotein lipase
PPARγ	peroxisome proliferator-activated receptor gamma
FAO	fatty acid oxidation
VLCFAs	very long-chain fatty acids
Fasn	fatty acid synthase
CPT1A	carnitine palmitoyltransferase 1A
ACSL4	long-chain acyl-coenzyme A synthase 4
CerS	ceramide synthase
ARG1	arginase 1
NO	nitric oxide
GLS	glutaminase
GS	glutamine synthetase
GLUD	glutamate dehydrogenase
α-KG	α-ketoglutarate
KMO	kynurenine 3-monooxygenase
3-HK	3-hydroxykynurenine
QA	quinolinic acid
NAD^+^	nicotinamide adenine dinucleotide
DRP1	dynamin-related protein 1
MFN	mitofusins
OPA1	optic atrophy 1
RET	reverse electron transport
SSRIs	selective serotonin reuptake inhibitors
SNRIs	serotonin–norepinephrine reuptake inhibitors
